# Pleiotropic Effects of Oral Anticoagulant Therapy: Is There a Difference Between VKAs and DOACs?

**DOI:** 10.3390/biomedicines13081850

**Published:** 2025-07-30

**Authors:** Francesco Alfano, Anna Maria Gori, Martina Berteotti, Angela Rogolino, Francesca Cesari, Emilia Salvadori, Benedetta Formelli, Francesca Pescini, Carmen Barbato, Betti Giusti, Anna Poggesi, Rossella Marcucci

**Affiliations:** 1 Department of Experimental and Clinical Medicine, University of Florence, 50134 Florence, Italy; annamaria.gori@unifi.it (A.M.G.); martina.berteotti@unifi.it (M.B.); rogolinoa@aou-careggi.toscana.it (A.R.); francesca.cesari@gmail.com (F.C.); betti.giusti@unifi.it (B.G.); rossella.marcucci@unifi.it (R.M.); 2 Center for Atherothrombotic Diseases, Careggi University Hospital, 50134 Florence, Italy; 3 Department of Biomedical and Clinical Sciences, University of Milan, 20157 Milan, Italy; emilia.salvadori@unimi.it; 4 NEUROFARBA Department, Neuroscience Section, University of Florence, 50134 Florence, Italy; benedetta.formelli@unifi.it (B.F.); francesca.pescini@unifi.it (F.P.); carmenbarbato88@gmail.com (C.B.); anna.poggesi@unifi.it (A.P.); 5 Stroke Unit, Careggi University Hospital, 50134 Florence, Italy

**Keywords:** atrial fibrillation, oral anticoagulant therapy, biomarkers, inflammation, hemostasis, extracellular matrix, vitamin K antagonist, direct oral anticoagulants, hypofibrinolysis

## Abstract

**Background**: Atrial fibrillation (AF) is one of the most common heart rhythm disorders encountered in clinical practice. Emerging evidence suggests a significant role of inflammation in the pathogenesis of AF, but certain questions still remain unanswered, in particular whether AF-related inflammation is a cause or a consequence of the arrhythmia, and whether inflammation reflects underlying disease or AF itself. At the current state of the art, scientific evidence on the role of oral anticoagulants (OAC) in modulating pro-inflammatory cytokines implicated in the pathogenesis of AF remains scarce. The aim of our study was to evaluate, in a population of AF patients undergoing OAC, the different roles of anticoagulant therapy [Vitamin K antagonists (VKAs) and direct oral anti-coagulants (DOACs)] in modulating the levels of inflammatory biomarkers in AF. **Methods**: The Strat-AF study is an observational, prospective, single center, hospital-based study enrolling elderly patients with AF. Results refer to 170 subjects with complete clinical and biohumoral assessment. **Results**: At multivariate logistic regression analysis, adjusted for several covariates, VKA treatment was an independent protective predictor for having a high grade of inflammation not balanced by anti-inflammatory cytokine levels [OR = 0.26 (0.10–0.69), *p* = 0.007]. **Conclusions**: These results from the Strat-AF study are “generators of hypotheses” and provide preliminary evidence for the differential effects of VKAs and DOACs on inflammatory biomarkers (e.g., IL-6, TNF-α) in AF patients. These findings suggest that inflammatory biomarkers could enhance stroke risk prediction models, potentially improving a tailored AF management.

## 1. Introduction

Atrial fibrillation (AF) is one of the most common heart rhythm disorders encountered in clinical practice [1]. It is associated not only with an increased risk of stroke and arterial thromboembolic events [2,3,4], but also with increased mortality [5], with up to a two-fold increased risk of all-cause mortality [6] and cardiovascular mortality [2] in AF patients compared with those on sinus rhythm. The use of oral anticoagulants (OACs) and their effectiveness to prevent ischemic stroke in patients with AF is well established [7]. However, despite the use of OACs, patients with a diagnosis of AF still have a residual risk of death [8].

The current thrombotic and bleeding risk stratification schemes strive to identify patients who may benefit from different types of OAC. The validity of such schemes relies solely on clinical information, whose reliability is debated and requires further refinement.

Emerging evidence suggests a meaningful role of inflammation and elevated levels of prothrombotic plasma markers in the pathogenesis of AF [9,10,11].

In this setting, certain questions still remain unanswered, in particular whether AF-related inflammation is a cause or a consequence of the arrhythmia, and whether inflammation reflects underlying disease or AF itself. A deeper understanding of the complex relationship between AF and inflammation is needed to determine whether inflammatory pathways can be targeted to enhance approaches to AF prevention and management. At the current state of the art, scientific evidence on the role of OACs in modulating pro-inflammatory cytokines remains scarce.

The aim of our study was to evaluate, in a population of AF patients undergoing OAC, the different role of anticoagulant therapy [Vitamin K antagonists (VKAs) and direct oral anti-coagulants (DOACs)] in modulating the levels of inflammatory biomarkers in AF.

We also hypothesized that VKAs, due to their broader effect on the coagulation cascade, may exert a greater anti-inflammatory effect than DOACs in AF patients.

## 2. Materials and Methods

### 2.1. Study Population

The design and methodology of the Strat-AF study (Stratifying cerebral bleeding risk in AF)—an observational, monocentric, prospective and hospital-based study enrolling elderly patients with AF—have been previously described [12].

The Strat-AF study was approved by the Ethics Committee of Careggi University Hospital on 14 March 2017 (project identification code 16RFAP).

For the aim of this paper, we collected the main demographic characteristics of the enrolled patients (age, sex and years of schooling) as well as the main cardiovascular risk factors and comorbidities (e.g., hypertension, diabetes, dyslipidemia, physical activity, smoking habit, alcohol consumption, peripheral arterial disease, ischemic heart disease, myocardial infarction and heart failure). All these characteristics were subsequently used for the statistical analysis.

### 2.2. Laboratory Determinations

Venous blood samples were collected in the afternoon by using tubes without anticoagulant and/or with citrate (3.2%, 0.109 M). All the determinations of the circulating biomarkers were performed in a unique central laboratory. After centrifugation of the blood samples at room temperature at 1500× *g* for 15 min, the supernatants were aliquoted and stored at −80 °C until biomarker assessment, which was performed, for all the samples, six months after the enrollment.

Serum inflammatory biomarker levels [interleukin (IL)-4, IL-6, IL-8, IL-10, Tumor Necrosis Factor alpha (TNF-α), chemokine (C-C motif) ligand 2 (CCL2), C-X-C motif chemokine ligand 10 (CXCL10), Intercellular Adhesion Molecule-1 (ICAM-1), Vascular Cell Adhesion Protein 1 (VCAM-1) and Vascular-Endothelial Growth Factor (VEGF)] and metalloproteinases [Matrix Metalloproteinase (MMP)-2, MMP-7, MMP-8, MMP-9, MMP-12, Extracellular Matrix Metalloproteinase Inducer (EMMPRIN), Tissue Inhibitor of Metalloproteinase (TIMP)-1, TIMP-2, TIMP-3 and TIMP-4] were determined using a Bio-Plex suspension array system (Bio-Rad Laboratories Inc., Hercules, CA, USA) and R&D Kits (R&D System, Milan, Italy). In all the assays, the coefficients of variation in the inflammatory biomarkers were less than 6%.

vWF and D-Dimer plasma levels were determined by a latex particle-enhanced immunoturbidimetric assay (Werfen, Milan, Italy). Circulating plasma levels of PAI-1 Antigen levels were assessed by an immunoenzymatic assay (Hyphen Biomed, Neuville-sur-Oise, France).

Thrombin generation was assessed in accordance with Hemker et al. [13] by using a calibrated automated thrombogram (CAT) and a Fluoroskan Ascent^®^ microplate fluorimeter (Thermo Fisher Scientific, Waltham, MA, USA). The parameters taken into account were the peak height (nM) of the CAT, the endogenous thrombin potential (ETP) (nM/min) [(=area under the curve), with and without thrombomodulin (TM) and their ratio] and, lastly, the time to peak (min) of the CAT.

The lysis of a tissue factor–induced clot by exogenous t-PA was determined with the method previously described by Lisman [14].

All measures were performed in duplicate.

### 2.3. Outcomes

The main outcome of the study was the evaluation of the grade of inflammation in patients treated—respectively—with VKAs vs. DOACs.

### 2.4. Statistical Analysis

All analyses were performed with SPSS 20.0 (SPSS Inc., Chicago, IL, USA) and Stata 13.0 (Lakeway Dr, College Station, TX, USA).

As a main explanatory variable, we used the baseline of inflammatory markers, MMPs and TIMPs, vWF and PAI-1 antigen levels, clot lysis time (CLT), and ETP (with and without TM and their ratio). Differences in these biomarker values were analyzed in relation to the type of OAC. Values are presented as median and interquartile range if they had a non-Gaussian distribution.

The Mann–Whitney U Test was chosen to analyze the main differences in the circulating biomarkers’ levels because of the relatively large statistical variations. The net effect of each biomarker’s baseline value on outcomes was then estimated by a logistic regression model, which included age, sex, CHA_2_DS_2_-VASc, HAS-BLED and the main traditional cardiovascular risk factors as covariates. These variables were chosen for the adjustment of the multivariate logistic regression analysis according to the significance in the univariate analysis. To correct the results for multiple comparisons, we used the false discovery rate (FDR) testing (Benjamini–Hochberg method) in all the statistical analyses. The significance level was defined as *p* < 0.05. We used false discovery rate adjustment as it is a less conservative approach to multiple comparisons correction than traditional methods (e.g., Bonferroni adjustment). An FDR value of 0.05 means that 5% of declared positive results are truly negative. If many *p* values fall into the range where the null hypothesis of no association should be rejected, the FDR is much less conservative. The FDR identifies a set of potential, or “candidate”, positive test results, more likely than other results, to be further investigated. The FDR controls are less conservative than in Bonferroni’s or Tuckey’s methods, leading to a lower false negative rate at the cost of a higher false positive rate. We chose the FDR in order to have a lower negative rate.

## 3. Results

The results refer to 170 patients (mean age 77.7 ± 6.8 years, females *n* = 59, 34.7%) enrolled in the Strat-AF Study. All the enrolled patients underwent a complete clinical and biohumoral assessment. The demographic and clinical characteristics of the cohort are shown in Table 1. A comparison of the demographic and clinical characteristics between the DOAC and VKA cohorts of the Strat-AF study is shown in Table 2.

All the subjects of the study were on OAC: 30.6% (*n* = 52) were on VKA, whereas 69.4% (*n* = 118) were on DOACs. In particular, with regard to the DOACs group, 41 (24.1%) were on apixaban, 18 (10.6%) on edoxaban, 24 (14.1%) on rivaroxaban, and 35 (20.6%) on dabigatran; in the VKA patients, 48 (28.2%) were treated with warfarin, whereas only 4 (2.4%) with acenocoumarol.

We also analyzed the prevalence of aspirin and statin use in the DOACs and VKA groups: 48 (28.2%) patients had a concomitant use of aspirin, whereas 58 (34.1%) had a concomitant use of statins. Aspirin use was significantly higher in patients on DOACs than in patients treated with VKAs [43 (36.8%) patients vs. 5 (9.6%) patients, *p* < 0.001). Similarly, patients on DOACs were more frequently treated with statins than patients on VKAs, even if statistical significance was not completely achieved [35 (29.7%) patients vs. 23 (44.2%) patients, *p* = 0.080].

Regarding patients on VKAs, 81.3% (*n* = 39) of them had an adequate TTR (>60%); meanwhile, for patients on DOACs, we assessed the circulating concentrations of DOACs using specific assays and found the majority of cases (>90%) to be in agreement with through values (apixaban 22–177 ng/mL; dabigatran 61–143 ng/mL; edoxaban 19–62 ng/mL; rivaroxaban 6–239 ng/mL). In order to evaluate the possible influence of DOAC concentrations on inflammatory biomarker levels, we performed a correlation analysis by using a Spearman non-parametric test, and we did not find any significant correlation between the DOAC concentrations and the inflammatory marker levels.

### 3.1. Circulating Biomarkers According to the Type of OAC

Patients treated with DOACs had higher circulating levels of IL-6, IL-10 and TNF-α [1.98 (1.42–3.58 vs. 0.38 (0.30–1.21), *p* < 0.001; 3.29 (1.09–3.56) vs. 1.45 (0.24–3.46), *p* = 0.003; 2.95 (1.51–5.00) vs. 1.53 (0.59–2.28), *p* < 0.001, respectively] if compared with the VKA group.

With regard to the adhesion molecules, patients treated with DOACs had higher levels of ICAM-1 and VCAM-1, but lower circulating levels of CXCL-10 if compared with the VKA group [343.62 (273.42–601.88) vs. 297.46 (247.84–385.63), *p* = 0.023; 1532.00 (1032.08–2148.13) vs. 1205.00 (971.11–1830.00), *p* = 0.036; 13.29 (9.85–20.54) vs. 16.65 (12.00–25.44), *p* = 0.043, respectively].

The analysis of clotting parameters showed that patients treated with VKAs had higher levels of PAI-1 and a higher ETP ratio (with/without thrombomodulin) but lower circulating levels of the “time to peak” parameter and ETP without thrombomodulin, compared to the DOACs group [11.33 (7.83–18.32) vs. 8.64 (6.83–12.53), *p* = 0.020; 0.87 (0.68–1.01) vs. 0.65 (0.41–0.90), *p* < 0.001; 10.80 (5.45–15.60) vs. 14.00 (8.70–20.70), *p* = 0.014; 448.45 (322.83–850.20) vs. 1242.00 (538.00–2417.40), *p* < 0.001, respectively].

Lastly, the analysis of the biomarkers of extracellular matrix remodeling showed that patients treated with VKAs had higher levels of MMP-7 and TIMP-3, but lower circulating levels of MMP-12, compared with patients treated with DOACs [6.90 (6.19–7.93) vs. 4.15 (2.35–5.71), *p* < 0.001; 46.11 (29.81–67.17) vs. 33.34 (22.96–49.24), *p* = 0.007; 79.35 (45.01–93.91) vs. 450.10 (309.12–612.28), *p* < 0.001, respectively] (Table 3).

In order to evaluate the role of the balance between anti-inflammatory and pro-inflammatory cytokines, we categorized patients into three groups, in order to create a score of inflammation:

- Group 1: patients in which the anti-inflammatory response overwhelms the pro-inflammatory cytokines (e.g., pro-inflammatory cytokines in the first or second tertiles and anti-inflammatory cytokines in the third tertile);
- Group 2: patients with a balanced anti-/pro-inflammatory cytokines ratio (e.g., pro-inflammatory and anti-inflammatory cytokines in the same tertiles);
- Group 3: patients with pro-inflammatory cytokines not compensated by anti-inflammatory cytokines (e.g., pro-inflammatory cytokines in the third tertile and anti-inflammatory cytokines in the first or second tertiles).

We have chosen to present data about the effect of different types of anticoagulants on the inflammatory score, as this score better represented the complex interplay between pro- and anti-inflammatory molecules in pathological processes. In fact, several cytokines involved in inflammatory processes have overlapping, antagonistic, and synergic effects on many cell types and up-regulate and down-regulate the production of other cytokines and inflammatory mediators. For these reasons, it is crucial to evaluate a full profile of pro-inflammatory and anti-inflammatory cytokines to obtain a more complete and precise picture of the evolving interaction between inflammation and the effect of anticoagulants [15].

In the first tertile (patients with low grade of inflammation), 17.8% (*n* = 21) were in anticoagulant therapy with DOACs and 28.8% (*n* = 15) were on anticoagulant therapy with VKAs. In the second tertile (patients with inflammation balanced by anti-inflammatory cytokines), 42.4% (*n* = 50) were on DOACs, and 53.8% (*n* = 28) on VKAs. In the third tertile (patients with a high grade of inflammation not balanced by anti-inflammatory cytokines), 39.8% (*n* = 47) were in anticoagulant therapy with DOACs and 17.3% (*n* = 9) were on anticoagulant therapy with VKAs (*p* = 0.013).

A graphical representation of the inflammatory score has been added in the Supplementary Material section (Figure S1).

### 3.2. Multivariate Analysis

In the multivariate logistic regression analysis adjusted for several covariates (age, sex, BMI, schooling years, hypertension, diabetes, dyslipidemia, smoking habit, lack of physical activity, previous stroke, coronary artery disease, heart failure, peripheral artery disease, CHA_2_DS_2_-VASs and HAS-BLED), VKA treatment was an independent protective predictor for having a high grade of inflammation not balanced by anti-inflammatory cytokine levels [OR = 0.26 (0.10–0.69), *p* = 0.007] (Table 4).

In the Supplementary Material section, we report the results of the multivariable linear regression analysis (Table S1) and give a graphical representation of the inflammatory markers by type of OAC (Figure S2).

## 4. Discussion

In the present study, we explored, in a cohort of AF patients under treatment with any type of oral anticoagulant, the role and contribution of anticoagulant therapy in reducing inflammation-related biomarkers in AF patients.

Our results showed a statistically significant reduction in circulating levels of pro-inflammatory cytokines in patients receiving VKA, suggesting a possible role of these pharmacological agents within the inflammatory pathways, which we know to be involved in the pathogenesis of AF. Recent studies have suggested a pleiotropic effect of DOACs on inflammation, but in our cohort of patients treated with DOACs, we failed to demonstrate a significant correlation between the inflammatory marker levels and the DOAC concentrations, suggesting that the smaller effect of the DOACs on inflammation with respect to that observed in patients treated with VKAs cannot be attributed to a lower concentration of DOACs.

In particular, the analysis of inflammation parameters in relation to the type of oral anticoagulant therapy has highlighted that patients treated with VKAs had significantly lower levels of IL-6 and TNF-α (pro-inflammatory cytokines) compared with the patients treated with DOACs. In the same group, the circulating levels of ICAM-1 and VCAM-1 were also significantly lower, while the levels of CXCL-10 (adhesion molecule with pro-inflammatory activity) were significantly higher in those taking VKAs.

IL-6 has been consistently identified as a key mediator in the pathophysiology of AF [16]. Emerging evidence suggests that higher circulating levels of IL-6 are significantly associated with an elevated risk of AF recurrence following cardioversion [17,18] and after radiofrequency catheter ablation [19], with poorer long-term outcomes, including greater risk of stroke and all-cause mortality [20]. Recent studies indicate that inhibiting IL-6 signaling can significantly reduce both the initiation and the persistence of AF episodes [21,22,23]. Elevated IL-6 levels seem to contribute to an inflammatory milieu that promotes atrial remodeling and atrial fibrosis, thereby exacerbating the underlying pathophysiological processes of AF by stimulating fibroblast proliferation and extracellular matrix deposition [24]. In animal models, IL-6 infusion increased ventricular stiffness and collagen deposition [25], whereas in isolated cardiac fibroblasts, the combination of IL-6 and soluble IL-6 receptors significantly increased collagen production: this promotes the transition of fibroblasts into myofibroblasts, further exacerbating atrial fibrosis and an excessive collagen deposition, which leads to the replacement of normal atrial tissue with fibrotic tissue [26,27]. This impairs atrial function and increases susceptibility to AF, especially under conditions where IL-6 levels are elevated [23,24,25,26,27,28].

Even TNF-α plays a crucial role in the pathogenesis of AF; elevated plasma levels of TNF-α have shown a positive correlation with left atrial diameter in patients with permanent AF [29]. A cohort study of 373 patients by Pinto et al. showed that higher circulating levels of TNF-α were significant predictors of ischemic stroke in patients with AF [30]. In AF patients, lymphomononuclear cells (the predominant immune cells that infiltrate the atrial myocardium) secrete high levels of TNF-α in the atrium, which can subsequently contribute to atrial fibrosis and electrical remodeling [31]. Moreover, TNF-α also increases the secretion of MMP-2 and MMP-9, which mediate atrial remodeling [32].

With regard to the analysis of the markers of hemostasis, patients treated with VKAs had higher ETP ratio values compared with the patients treated with DOACs. A recent study by Dirienzo et al. showed that dabigatran (but no anti-Xa drugs) reduced ETP values in 137 patients with AF (*n* = 72) or venous thromboembolism (VTE) (*n* = 65) undergoing OAC with DOACs [33]. The ratio between ETP measured in the presence and absence of thrombomodulin is an index of hypercoagulability (in case of ratio > 0.8): these data are noteworthy and warrant further targeted investigation, also considering the well-known cerebral tropism of warfarin’s hemorrhagic complications.

Moreover, VKA patients have significantly higher levels of PAI-1, suggesting a condition of hypercoagulability (in harmony with the increased levels of the ETP ratio) resulting from hypofibrinolysis. This last finding is in agreement with what has already been described in the literature in a small cohort of patients on oral anticoagulant therapy with warfarin [34], confirming how hypofibrinolysis plays a pivotal role in patients suffering from AF [35,36].

Lastly, the finding of higher levels of MMP-7 and TIMP-3 in patients treated with VKA, as well as the reduced circulating levels of MMP-12, corroborates existing evidence showing impaired extracellular matrix (ECM) degradation in AF patients. Matrix-degrading proteinases have previously been associated with left atrial dilatation and may serve as indirect markers of the overall burden of cardiac fibrosis and cardiomyopathy [37]. However, data on the role of MMP-7 remain limited. Wang and colleagues investigated the role of MMP-7 and AF through an immunohistochemical analysis on atrial tissue collected from patients undergoing artificial mitral valve replacement surgery, showing that MMP-7 levels were significantly different between AF patients and controls in tissue mRNA, but not in the circulating protein [38]. Regarding TIMP-3, there is no evidence linking its circulating levels to an increased AF risk [37].

Our results are “generators of hypotheses” for understanding the possible pleiotropic role of oral anticoagulant therapy with VKAs in AF patients. Currently, several works have demonstrated the role of the inflammatory pathways in the pathogenesis of this common arrhythmia, especially as regards atrial remodeling and alteration of the cardiac electrical conduction network, but at the current state of the art, scientific evidence on the role of OAC in modulating pro-inflammatory cytokines implicated in the pathogenesis of AF remains scarce and is limited to the use of rivaroxaban [39], whose safety and efficacy have already been proved in a cohort of AF patients undergoing catheter ablation [40]. Identifying a possible effect of oral anticoagulant therapy on these pathways would pave the way to further possible scenarios of clinical relapse. A recent mendelian randomization by Hu et al. showed a causal association between genetically predicted levels of circulating cytokines and the risk of VTE, suggesting how strategies targeting circulating cytokines may act as a prevention approach [41].

A possible explanation for these results could lie in the larger and more extensive effect of VKA in the coagulation cascade, if compared to DOACs which are single-target drugs. VKA, blocking vitamin K epoxide reductase, decreases the plasma concentrations of clotting factors II, VII, IX, and X. It is known that vitamin K-dependent clotting factors play a pivotal role in inflammation and can themselves initiate the production of pro-inflammatory mediators, including IL-6 and IL-8 [42,43].

Recently, the mechanism of thrombogenesis in AF has ventured into new areas of research with the involvement of neutrophil extracellular traps (NETs) [44]. NETs may constitute useful thrombogenesis risk markers in AF patients and provide a potential therapeutic strategy for AF management, as they lie at the interface of inflammation, thrombosis, and fibrosis [45], being dynamic players that actively participate in various aspects of the thrombotic process [46,47].

However, our study has some limitations. Firstly, the study population was carefully selected according to the inclusion criteria of the study; in particular, we enrolled patients ≥ 65 years, as AF is typically a condition affecting the elderly, and its incidence and prevalence progressively increase with aging, particularly from the age of 65 years onward.

Secondly, these findings might also be influenced by other ongoing therapies in the population study.

Lastly, the relatively small sample size of the study limited the generalizability of our findings to the broader population of AF patients. The imbalance between the number of patients treated with DOACs and those treated with VKAs represents an additional limitation. However, the Strat-AF Study enrolled patients from a “real-world” setting, where the proportion of patients on VKAs is typically low. Further studies involving a larger AF population and ensuring a more balanced representation of the DOAC- and VKA-treated patients are needed to confirm these preliminary findings and to strengthen the statistical power of the analysis.

## 5. Conclusions

In conclusion, these findings from the Strat-AF study provide preliminary evidence for the differential effects of VKAs and DOACs on inflammatory biomarkers in AF patients, warranting further investigation. Although further research is warranted and necessary to validate these preliminary findings and to explore the underling biological mechanisms, these results might be a first step towards the incorporation of such new markers in existing stroke prediction schemes by demonstration of the greater incremental value of a tailored approach in predicting stroke risk, ultimately improving patient care.

## Figures and Tables

**Table 1:** Demographic and clinical characteristics of the baseline Strat-AF study cohort (*n* = 170). Results are expressed as median ± DS and as percentage.

Demographic and Clinical Characteristics	Total Cohort *n* = 170
Age [yrs], (mean ± SD)	77.7 ± 6.8
Female sex, *n* (%)	59 (34.7%)
Schooling [yrs], (mean ± SD)	9.1 ± 4.3
Stroke, *n* (%)	38 (22.4%)
Coronary artery disease, *n* (%)	18 (10.6%)
Heart failure, *n* (%)	25 (14.7%)
Peripheral arterial disease, *n* (%)	14 (8%)
Hypertension, *n* (%)	140 (82.4%)
Diabetes, *n* (%)	22 (12.9%)
Dyslipidemia, *n* (%)	87 (51.2%)
Physical activity (lack of), *n* (%)	110 (64.7%)
Smoking habit [current or former(within 10 yrs)], *n* (%)	105 (61.8%)
Alcohol consumption, *n* (%)	91 (53.5%)
BMI [kg/m^2^], (mean ± SD)	26.3 ± 3.9
CHA_2_DS_2_-VASc Score (mean ± SD)	3.69 ± 1.49
HAS-BLED (mean ± SD)	1.85 ± 0.89

**Table 2:** Comparison of the demographic and clinical characteristics between the DOAC and VKA cohorts of the Strat-AF study.

	Total Cohort (*n* = 170)	DOACs (*n* = 118)	VKAs (*n* = 52)	*p*
Age [yrs], (mean ± SD)	77.7 ± 6.8	77.5 ± 3.9	78.2 ± 6.4	0.512
Sex (M/F), *n* (%)	**111/59**	**70 (59.3)/48 (40.7)**	**41 (78.8)/11 (21.2)**	**0.015**
BMI [kg/m^2^], (mean ± SD)	26.3 ± 3.9	26.1 ± 3.9	26.9 ± 3.8	0.974
Schooling [yrs], (mean ± SD)	9.1 ± 4.3	9.3 ± 4.4	9.6 ± 4.3	0.417
Hypertension, *n* (%)	140 (82.4)	99 (83.9)	41 (78.8)	0.513
Diabetes, *n* (%)	22 (12.9)	13 (11.0)	9 (17.3)	0.321
Dyslipidemia, *n* (%)	87 (51.2)	55 (46.6)	32 (61.5)	0.096
Smoking habits, *n* (%)	105 (61.8)	70 (59.3)	35 (67.3)	0.393
Physical activity (lack of), *n* (%)	110 (64.7)	75 (63.6)	35 (67.3)	0.728
Alcohol consumption, *n* (%)	91 (53.5%)	57 (48.3)	34 (65.4)	0.046
Stroking habit, *n* (%)	38 (22.4)	27 (22.9)	11 (21.1)	0.845
Coronary artery disease, *n* (%)	18 (10.6)	11 (9.3)	7 (13.5)	0.427
Heart failure, *n* (%)	**25 (14.7)**	**9 (7.6)**	**16 (30.8)**	**<0.001**
Peripheral arterial disease, *n* (%)	14 (8)	6 (5.1)	8 (15.4)	0.034
CHA_2_DS_2_-VASc (mean ± SD)	3.7 ± 1.5	3.7 ± 1.4	3.8 ± 1.5	0.704
HAS-BLED (mean ± SD)	**1.9 ± 0.9**	**1.6 ± 0.7**	**2.1 ± 1.0**	**0.012**
Aspirin use, *n* (%)	**48 (28.2)**	**43 (36.8)**	**5 (9.6)**	**<0.001**
Statin use, *n* (%)	58 (34.1)	35 (29.7)	23 (44.2)	0.080

**Table 3:** Circulating biomarkers in relation to the type of OAC. Results are expressed as median [(interquartile range (IQR)].

Biological Markes	Type of OAC		
	DOACs (*n* = 118)	VKAs (*n* = 52)	*p*
IL-4 [pg/mL], median (IQR)	12.81 (5.00–35.92)	6.50 (6.00–15.54)	0.067
IL-6 [pg/mL], median (IQR)	**1.98 (1.42–3.58)**	**0.38 (0.30–1.21)**	**<0.001**
IL-8 [pg/mL], median (IQR)	8.18 (4.70–12.10)	9.44 (5.77–14.32)	0.180
IL-10 [pg/mL], median (IQR)	**3.29 (1.09–3.56)**	**1.45 (0.24–3.46)**	**0.003**
TNFα [pg/mL], median (IQR)	**2.95 (1.51–5.00)**	**1.53 (0.59–2.28)**	**<0.001**
CCL-2 [pg/mL], median (IQR)	328.79 (230.65–465.40)	309.78 (228.15–380.14)	0.153
CXCL-10 [pg/mL], median (IQR)	**13.29 (9.85–20.54)**	**16.65 (12.00–25.44)**	**0.043**
ICAM-1 [ng/mL], median (IQR)	**343.62 (273.42–601.88)**	**297.46 (247.84–385.63)**	**0.023**
VCAM-1 [ng/mL], median (IQR)	**1532.00 (1032.08–2148.13)**	**1205.00 (971.11–1830.00)**	**0.036**
VEGF [pg/mL], median (IQR)	66.03 (37.46–112.81)	61.21 (35.24–85.75)	0.454
PAI-1 [ng/mL], median (IQR)	**8.64 (6.83–12.53)**	**11.33 (7.83–18.32)**	**0.020**
vWF [%], median (IQR)	152.50 (122.20–211.70)	178.50 (139.20–205.90)	0.169
Peak [nM], median (IQR)	90.50 (29.80–231.15)	60.65 (37.25–146.40)	0.233
Time to peak [min], median (IQR)	**14.00 (8.70–20.70)**	**10.80 (5.45–15.60)**	**0.014**
ETP TM- [nM/min], median (IQR)	687.00 (243.00–1835.40)	387.25 (291.38–752.88)	0.066
ETP TM + [nM/min], median (IQR)	**1242.00 (538.00–2417.40)**	**448.45 (322.83–850.20)**	**<0.001**
ETP ratio [ratio], median (IQR)	**0.65 (0.41–0.90)**	**0.87 (0.68–1.01)**	**<0.001**
Clot lysis time [min], median (IQR)	53.82 (44.56–72.14)	50.24 (40.76–59.04)	0.088
EMMPRIN [ng/mL], median (IQR)	5.44 (3.84–6.88)	5.54 (4.12–6.91)	0.870
MMP-2 [ng/mL], median (IQR)	521.51 (438.74–640.58)	496.31 (415.28–628.78)	0.260
MMP-7 [ng/mL], median (IQR)	**4.15 (2.35–5.71)**	**6.90 (6.19–7.93)**	**<0.001**
MMP-8 [ng/mL], median (IQR)	7.07 (3.43–14.61)	8.32 (4.28–13.66)	0.538
MMP-9 [ng/mL], median (IQR)	319.50 (195.74–513.62)	269.25 (176.34–412.66)	0.138
MMP-12 [ng/mL], median (IQR)	**450.10 (309.12–612.28)**	**79.35 (45.01–93.91)**	**<0.001**
TIMP-1 [ng/mL], median (IQR)	162.20 (131.25–201.62)	164.24 (135.69–238.15)	0.690
TIMP-2 [ng/mL], median (IQR)	126.78 (99.12–161.87)	140.62 (98.01–220.67)	0.363
TIMP-3 [ng/mL], median (IQR)	**33.34 (22.96–49.24)**	**46.11 (29.81–67.17)**	**0.007**
TIMP-4 [ng/mL], median (IQR)	3.08 (2.36–4.25)	3.14 (2.14–5.67)	0.874

**Table 4:** Univariate and multivariate analysis for type of OAC.

	Univariate Analysis	*p*	Multivariate Analysis OR (95% CI)	*p*
	OR (95% CI)			
**Age (yrs)**				
Balance	1.02 (0.96–1.08)	0.477		
High grade	1.02 (0.96–1.09)	0.551		
**Sex, F vs. M**				
Balance	0.79 (0.34–1.81)	0.572		
High grade	1.23 (0.52–2.93)	0.634		
**BMI [kg/m2]**				
Balance	0.95 (0.86–1.05)	0.311		
High grade	0.97 (0.87–1.08)	0.574		
**Schooling [yrs]**				
Balance	1.13 (1.02–1.25)	0.020		
High grade	1.09 (0.98–1.21)	0.107		
**Hypertension**				
Balance	0.36 (0.11–1.15)	0.086	0.41 (0.13–1.33)	0.136
High grade	1.04 (0.27–3.98)	0.952	1.10 (0.28–4.34)	0.897
**Diabetes**				
Balance	1.18 (0.34–4.04)	0.796		
High grade	1.33 (0.37–4.80)	0.660		
**Dyslipidemia**				
Balance	0.55 (0.25–1.23)	0.145		
High grade	0.95 (0.41–2.22)	0.910		
**Smoking habit**				
Balance	1.36 (0.61–3.00)	0.453		
High grade	2.05 (0.86–4.89)	0.104		
**Physical activity (lack of)**				
Balance	0.83 (0.36–1.942)	0.669		
High grade	0.68 (0.28–1.65)	0.395		
**Alcohol consumption**				
Balance	1.34 (0.61–3.00)	0.453		
High grade	0.72 (0.31–1.67)	0.722		
**Stroke**				
Balance	0.50 (0.20–1.24)	0.134		
High grade	0.69 (0.27–1.76)	0.453		
**Coronary artery disease**				
Balance	1.26 (0.31–5.05)	0.104		
High grade	1.57 (0.38–6.52)	0.388		
**Heart failure**				
Balance	0.54 (0.18–1.59)	0.263		
High grade	0.79 (0.27–2.36)	0.677		
**Peripheral arterial disease**				
Balance	1.28 (0.31–5.05)	0.747		
High grade	0.62 (0.12–3.27)	0.575		
**CHA2DS2-VASc**				
Balance	0.82 (0.63–1.07)	0.149		
High grade	0.96 (0.73–1.26)	0.766		
**HAS-BLED**				
Balance	0.79 (0.50–1.25)	0.321		
High grade	0.83 (0.51–1.35)	0.453		
**Type of OAC** **(VKAs vs. DOACs)**				
Balance	0.78 (0.35–1.76)	0.069	0.73 (0.32–1.67)	0.453
High grade	**0.27 (0.10–0.71)**	**0.008**	**0.26 (0.10–0.69)**	**0.007**
**Aspirin use**				
Balance	0.63 (0.26–1.54)	0.314		
High grade	1.29 (0.53–3.19)	0.568		
**Statin use**				
Balance	0.52 (0.23–1.19)	0.118		
High grade	0.91 (0.39–2.12)	0.820		

## Data Availability

The data presented in this study are available on request from the corresponding author. The data are not publicly available due to privacy or ethical restrictions.

## Supplementary Materials

The following supporting information can be downloaded at: https://www.mdpi.com/article/10.3390/biomedicines13081850/s1, Figure S1: Graphical representation of the inflammatory score; Table S1: Multiple linear regression models: type of anticoagulant (VKA vs DOAC) effects on the circulating inflammatory markers levels (Beta ± SE and 95% CI); Figure S2: Graphical representation of the inflammatory markers by type of OAC (Box-plot with median and interquartile ranges).
