# Supplementary material for: Pleiotropic Effects of Oral Anticoagulant Therapy: Is There a Difference Between VKAs and DOACs?
Source: Biomedicines. 2025 Jul 30;13(8):1850. doi: 10.3390/biomedicines13081850 (PMC12383677; doi:10.3390/biomedicines13081850)

Figure S1: Graphical representation of the inflammatory score.

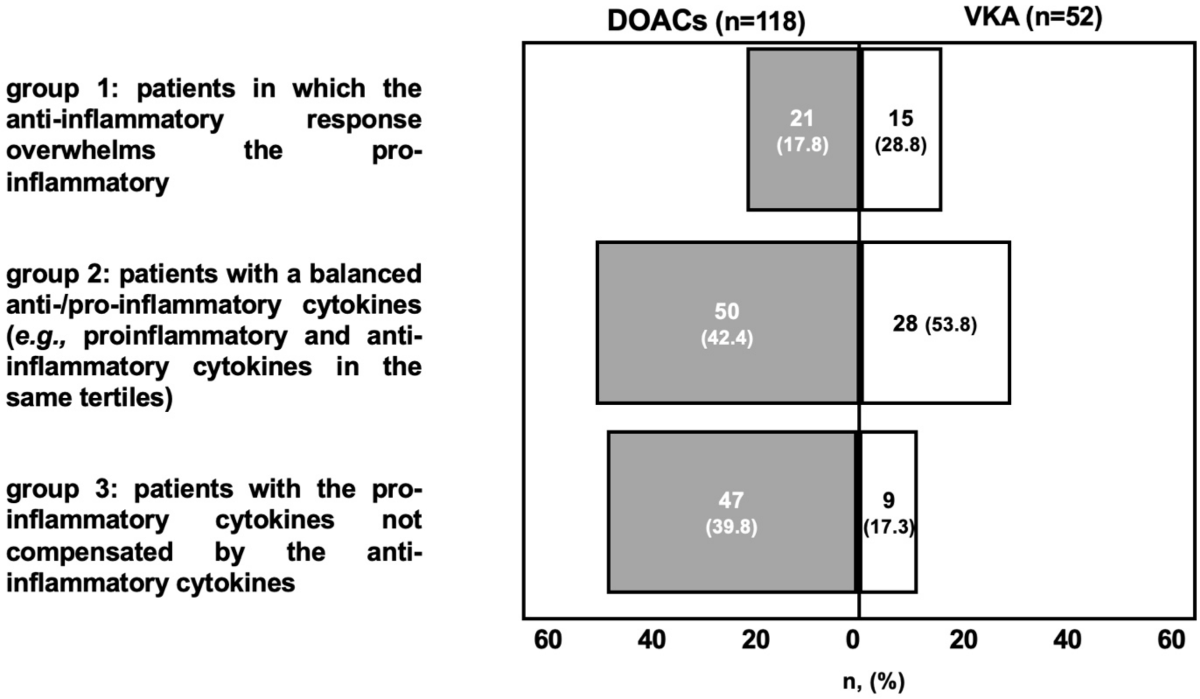

Table S1: Multiple linear regression models: type of anticoagulant (VKAs vs DOAC) effects on circulating inflammatory markers levels (Beta± SE and 95% CI).

	**Beta ± SE**	**95% CI**	**p**
**Log (IL-4):**			
**VKA vs DOAC**	**-0.213 ± 0.083**	**-0.396 / -0.069**	**0.006**
**Log (IL-6):**			
**VKA vs DOAC**	**-0.424 ± 0.081**	**-0.647 / - 0.327**	**0.001**
**Log (IL-8):**			
**VKA vs DOAC**	0.102 ± 0.055	-0.036 / 0.018	0.102
**Log (IL-10):**			
**VKA vs DOAC**	**-0.271 ± 0.093**	**-0.515 / - 0.150**	**0.001**
**Log (TNF-alpha):**			
**VKA vs DOAC**	-0.252 ± 0.092	-0.475 / -0.113	0.002
**Log (CCL-2):**			
**VKA vs DOAC**	-0.066 ± 0.044	-0.121 / 0.054	0.448
**Log (CXCL-10):**			
**VKA vs DOAC**	0.135 ± 0.046	-0.012 / 0.077	0.097
**Log (ICAM-1):**			
**VKA vs DOAC**	-0.193 ± 0.046	-0.201 /-0.021	0.026
**Log (VCAM-1):**			
**VKA vs DOAC**	**-0.189 ± 0.073**	**-0.318 /-0.030**	**0.018**
**Log (VEGF):**			
**VKA vs DOAC**	-0.134 ± 0.056	-0.203 / 0.016	0.093

Backward Regression Model adjusted for age, sex, BMI, physical activity (lack of), schooling years, hypertension, diabetes, dyslipidemia, smoking habit, CHA_2DS_2-VASc, HAS-BLED, peripheral artery disease, previous stroke, coronary artery disease, heart failure, peripheral artery disease, statin and aspirin treatment.

Figure S2: Graphical representation of the inflammatory markers by type of OAC (Box-plot with median and interquartile ranges).

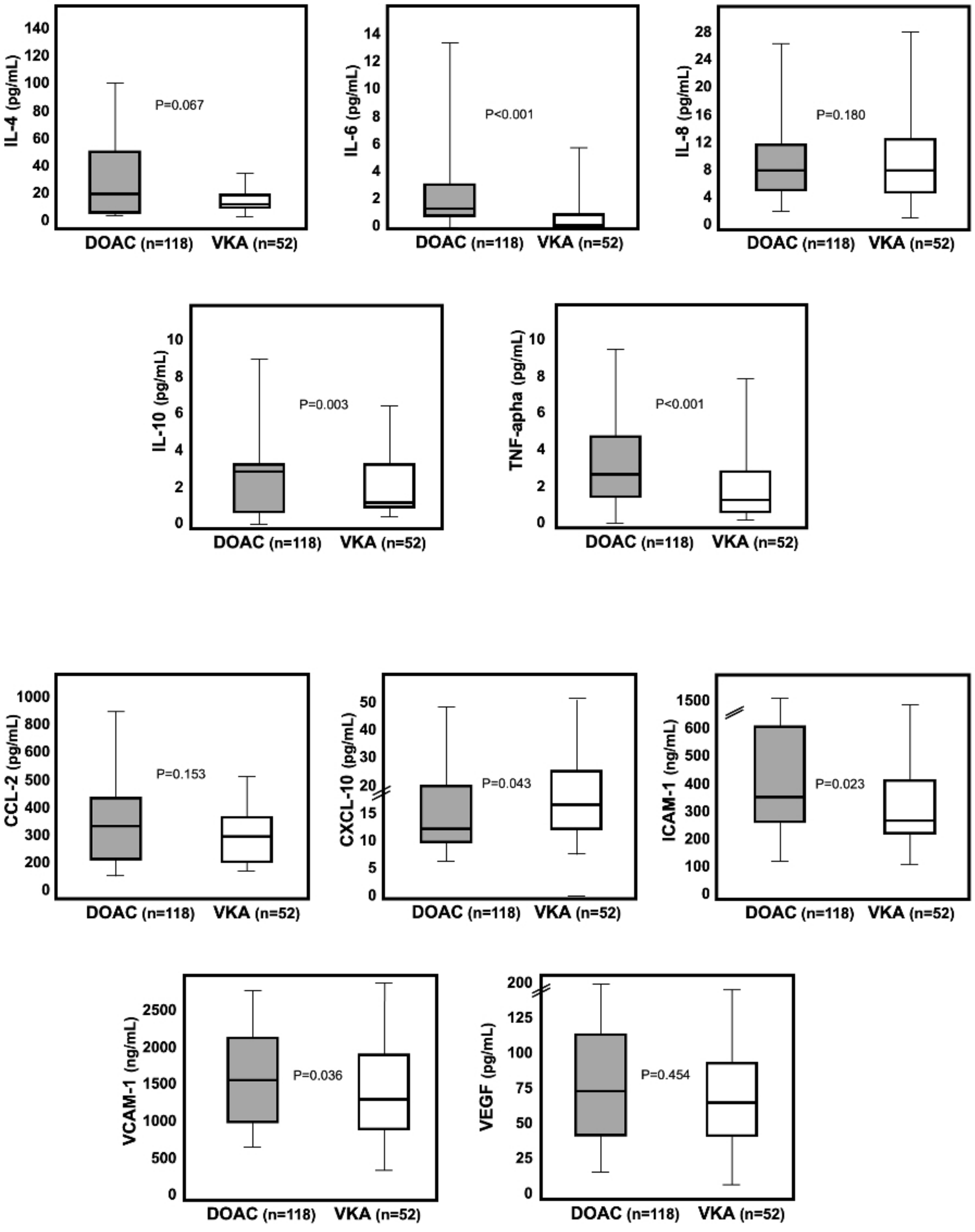

Supplement: Supplementary file 1 [file biomedicines-13-01850-s001.zip › biomedicines-3704464-supplementary.pdf]
